# P24 Scale and spread of a metronidazole ‘Go Green’ IV to oral switch (IVOST) grassroots quality improvement initiative in Scotland’s largest acute health board

**DOI:** 10.1093/jacamr/dlaf118.031

**Published:** 2025-07-14

**Authors:** R Rodger, A Robertson, S Thompson, E Thompson, K Downie, G Ray, L Chisholm, C Pender, K Philips, M Neto, R Hillson, R A Seaton, S Carty, K MacFarlane, N MacDonald, F Kinnaird, A Reuben, S Gillen, Y Gourlay, F Robb, L Stewart, R Puckett, M MacLeod

**Affiliations:** NHS Greater Glasgow and Clyde, Glasgow, UK; NHS Greater Glasgow and Clyde, Glasgow, UK; NHS Greater Glasgow and Clyde, Glasgow, UK; NHS Greater Glasgow and Clyde, Glasgow, UK; NHS Greater Glasgow and Clyde, Glasgow, UK; NHS Greater Glasgow and Clyde, Glasgow, UK; NHS Greater Glasgow and Clyde, Glasgow, UK; NHS Greater Glasgow and Clyde, Glasgow, UK; NHS Greater Glasgow and Clyde, Glasgow, UK; NHS Greater Glasgow and Clyde, Glasgow, UK; Centre for Sustainable Healthcare, UK; NHS Greater Glasgow and Clyde, Glasgow, UK; NHS Greater Glasgow and Clyde, Glasgow, UK; NHS Greater Glasgow and Clyde, Glasgow, UK; NHS Greater Glasgow and Clyde, Glasgow, UK; NHS Greater Glasgow and Clyde, Glasgow, UK; NHS Greater Glasgow and Clyde, Glasgow, UK; NHS Greater Glasgow and Clyde, Glasgow, UK; NHS Greater Glasgow and Clyde, Glasgow, UK; NHS Greater Glasgow and Clyde, Glasgow, UK; NHS Greater Glasgow and Clyde, Glasgow, UK; NHS Greater Glasgow and Clyde, Glasgow, UK; NHS Greater Glasgow and Clyde, Glasgow, UK

## Abstract

**Background:**

Promoting appropriate IV to oral switch (IVOST)^1^ is a fundamental antimicrobial stewardship (AS) initiative providing benefits for patients, staff and the environment including: reduced risk of cannula related infections, medicine costs, nursing workload and carbon footprint.^1,2^ A grassroots quality improvement (QI) initiative, highlighting the high oral bioavailability (>90%) of metronidazole and environmental benefits of IVOST in surgical wards at the Royal Alexandra Hospital (RAH), resulted in a 45% median reduction in IV administrations.^3^ Scale and spread of successful small scale QI initiatives,^4^ although challenging in the complex healthcare environment, is essential to maximize patient benefits and support attainment of national targets for antimicrobial resistance (AMR)^5^ and environmental sustainability.^6^

**Objectives:**

This study set out to scale and spread the QI initiative promoting metronidazole IVOST to all acute hospitals in NHSGGC, the largest acute health board in Scotland, and measure the impact on AS, drug costs, nursing workload and environmental sustainability.

**Methods:**

A QI scale and spread approach was used including the following. Eye catching posters displayed in key ward areas/electronic guideline platform. Electronic prescribing (HEPMA) IVOST prompts displayed when prescribing or administering IV metronidazole. ‘Think tabs before jags’ blog^7^ was used to highlight the initiative widely. HEPMA targeted prospective audit/feedback and antimicrobial ward round review of patients prescribed IV metronidazole. Multidisciplinary staff champions and Antibiotic Guardian ‘Gold Star’ awards were used to promote staff engagement. Regular feedback to medical, nursing and pharmacy teams via update reports and hospital governance committees. Oral and IV metronidazole usage data was calculated at baseline and for 40 months ‘post change’. Improvement measures in terms of reduced IV administrations, drug costs and nursing time saved^8^ were calculated. Plastic waste reduction (giving sets, single use plastics, cannula, safety needles, gloves and aprons) associated with IVOST was calculated in terms of carbon dioxide equivalent (CO2e) emissions using a hybrid carbon foot-printing methodology and emissions factor databases.^9^

**Results:**

A 33% median reduction in total (IV plus oral) metronidazole defined daily doses (DDDs) per 1000 occupied bed days (OBDs) (Figure 1). A 46% median reduction in IV metronidazole DDDs/1000 OBDs (Figure 2), equating to 5250 and 63 000 fewer IV administrations monthly and annually, respectively. Equivalent nursing time saved equating to 1750 h per month and 21 000 h per year. A 37% (£60 000) annual reduction in metronidazole drug cost. The carbon footprint saving achieved from metronidazole IVOST was calculated as 1.48111 KgCO2e per dose equating to 7775 KgCO2e per month and an annual carbon footprint saving of 93.3 tonnes CO2e.

**Conclusions:**

A QI scale and spread approach, to raise awareness of the multidisciplinary team to the high oral bioavailability of metronidazole and benefits of appropriate IVOST, resulted in a change in prescribing behaviour and a significant reduction in IV administrations. The initiative also resulted in a sustained reduction in total metronidazole use across NHSGGC acute hospitals. This is important in terms of improved AS, patient safety, workforce cost and efficiency and environmental sustainability. Crucially these results support attainment of national targets for tackling AMR^5^ and the climate emergency.^6^

**Figure 1.**
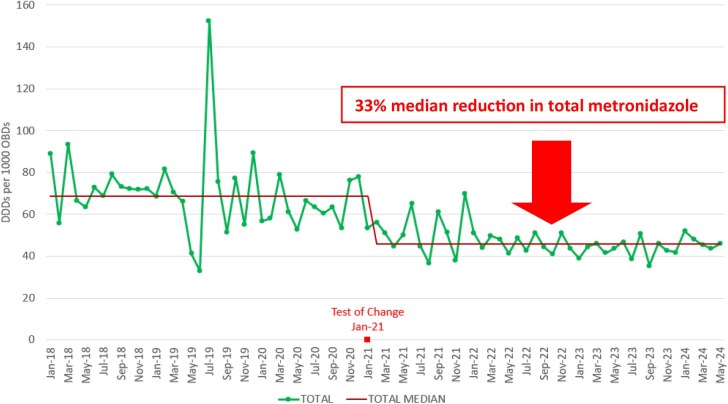
Total metronidazole (IV+ oral) DDDs per 1000 OBDs.

**Figure 2.**
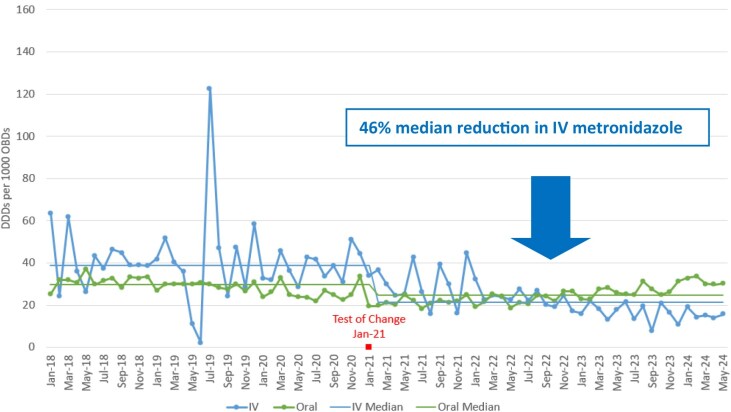
IV metronidazole and oral metronidazole DDDs per 1000 OBDs.
